# The direct response of the gonads to cues of stress in a temperate songbird species is season-dependent

**DOI:** 10.7717/peerj.139

**Published:** 2013-08-15

**Authors:** Nicolette L. McGuire, Annie Koh, George E. Bentley

**Affiliations:** 1 Laboratory of Reproductive Neuroendocrinology, Department of Integrative Biology, University of California at Berkeley, USA; 2 Helen Wills Neuroscience Institute, University of California at Berkeley, Berkeley, CA, USA

**Keywords:** Stress, Gonadotropin inhibitory hormone (GnIH), Corticosterone, Gonadotropin releasing hormone (GnRH), Testes, Estradiol, Testosterone, Avian, Supplementary cues, Photoperiod, Ovary

## Abstract

The gonadotropin releasing hormone (GnRH) system in the hypothalamus is often considered the final point in integration of environmental cues as they pertain to the reproductive axis. However, cues such as stress and food availability are detectable in the plasma (as glucocorticoid and metabolic fuel fluctuations). Vertebrate gonads express glucocorticoid receptor, therefore we hypothesized that the gonads can detect and respond directly to cues of stress. We provide evidence here that, in addition to regulation by the brain, the gonads of European starlings (*Sturnus vulgaris*) respond directly to fluctuations in corticosterone and metabolic fuels by modulating sex steroid secretion. Using a 4-h gonad culture, we show that physiologically-relevant concentrations of corticosterone and metabolic stress (via use of the glucose utilization inhibitor 2-deoxy-D-glucose and the fatty acid oxidation inhibitor ethyl 2-mercaptoacetate (2DG/MA)) can directly decrease testosterone and estradiol secretion from luteinizing hormone and follicle-stimulating hormone (LH/FSH)-stimulated testes and ovaries. This effect is regulated seasonally. Prior to the breeding season, testes and ovaries respond to corticosterone and 2DG/MA by significantly decreasing gonadal steroid release. Within the breeding season, the testes do not respond to these cues of stress, while the ovaries respond only to corticosterone. This seasonal difference in response may be due in part to the influence of these cues of stress on gonadal neuropeptide expression: corticosterone upregulates GnIH expression in the testes while metabolic stress upregulates GnIH in the ovaries. Thus the gonads can directly respond to fluctuations in corticosterone and metabolic fuels during a time of critical importance to the onset of breeding.

## Introduction

Many seasonal breeders undergo seasonal cycles of gonadal growth and regression, accompanied by changes in sex steroid production and gamete maturation. These cycles are modulated primarily by photoperiod cues (Dawson et al., 2001; Nicholls, Goldsmith & Dawson, 1988). Despite the strong influence of changing day length on the timing of reproduction, the precise timing of the onset of breeding can be modulated by multiple supplementary cues: food availability, temperature, weather, social cues and sustained stress (Gibb, 1950; Wingfield, 1984; Wingfield, 1985a; Wingfield, 1985b; Wingfield, Moore & Farner, 1983). Traditionally, release or inhibition of the neuropeptide gonadotropin-releasing hormone (GnRH) from the hypothalamus has been considered as the final integration point of photoperiodic and supplemental information (Ball, 1993). However, the appropriate timing of reproduction and synchrony with conspecifics is so important that multiple regulatory points in the hypothalamo-pituitary-gonad (HPG) axis might well be required.

Gonadotropin inhibiting hormone (GnIH) is another hypothalamic neuropeptide that modulates the secretion of luteinizing hormone (LH) and follicle stimulating hormone (FSH) from the anterior pituitary gland; it also inhibits action of GnRH neurons directly (Bentley et al., 2006; Bentley et al., 2009; Ducret, Anderson & Herbison, 2009; Tsutsui et al., 2000; Ubuka et al., 2008). Both GnRH and GnIH in the brain are altered in response to food restriction and stimulation of the hypothalamo-pituitary-adrenal (stress) axis (Bergendahl, Perheentupa & Huhtaniemi, 1991; Calisi, Perfito & Bentley, 2010; Ciccone, Dunn & Sharp, 2007; Grieves et al., 2008; Kirby et al., 2009; Rivest, Plotsky & Rivier, 1993). Recently, GnIH and GnIH receptor (GnIHR) expression were identified in the gonads of songbirds and primates, in addition to the brain (Bentley et al., 2008; McGuire & Bentley, 2010b). Furthermore, GnIH was able to decrease directly and dose-dependently the amount of testosterone secreted by gonadotropin-stimulated house sparrow testes *in vitro* (McGuire & Bentley, 2010a). The gonadal GnIH system can be modulated directly by circulating melatonin, thereby influencing gonadal steroid release independently of the brain (McGuire, Kangas & Bentley, 2011). Gonadotropin releasing hormone (GnRH) and its receptor (GnRHR) are also expressed in the gonads of vertebrates and have direct and typically inhibitory effects on sex steroid synthesis in species from protochordates to mammals, also the way in which the gonadal GnRH system is modulated is less clear (McGuire & Bentley, 2010b).

Vertebrate gonads are physiologically capable of detecting some stress cues. Plasma corticosterone and cortisol vary in response to stress in a season-specific manner (Romero, 2002) and receptors for glucocorticoids are present in gonadal tissues of songbirds (Kwok et al., 2007; Lattin et al., 2011) and other vertebrates (Bambino & Hsueh, 1981; Denari & Ceballos, 2006; Hsueh & Erickson, 1978; Kwok et al., 2007). Acute stress can also decrease plasma testosterone in a wild male songbird, apparently in the absence of a reduction in plasma LH (Deviche et al., 2010), indicating a potentially direct effect of stress on the gonads. This effect can persist for up to 6 h (Deviche et al., 2012). In addition, food restriction can be detected in the plasma through fluctuations in metabolic fuels, and can have an effect on reproduction (Szymanski et al., 2007). As metabolic fuels are required for gamete and sex steroid production, it is likely that the gonads detect these fluctuations in plasma glucose and fatty acids directly in addition to fluctuations in circulating glucocorticoids.

As a result of the fact that mammalian and avian gonads express glucocorticoid receptors, and because food restriction can inhibit reproduction and sexual behavior (Schneider, 2004), we sought to determine whether gonads of European starlings (*Sturnus vulgaris*) decrease sex steroid secretion in direct response to exposure to glucocorticoids or reduced access to metabolic fuel, via the gonadal GnIH and GnRH systems.

## Materials and Methods

All procedures were performed in accordance with federal and state laws and with appropriate agreements from the UC Berkeley Office of Laboratory Animal Care.

European starlings (*Sturnus vulgaris*) housed under natural photoperiods in outdoor aviaries in Berkeley, CA were captured in mist and hand nets on February 5, 2010 (*n* = 6 males, 6 females) and April 7, 2010 (*n* = 6 males, 5 females). February 5, 2010 had a day length of 10 h 27 min and was preceded by >6 weeks of natural short days. April 7, 2010 had a day length of 12 h 52 min. All birds were terminally anesthetized using isoflurane and then decapitated within 3 min of capture.

### Gonad culture

Gonad culture was performed on each day of capture with an identical procedure, except where noted. Testes and ovaries from each bird were removed, measured and incubated in individual tubes of Dulbecco’s modified eagle medium (DMEM; Sigma cat# D8437) for 4 h at 4°C prior to experimentation to establish basal levels of steroidogenesis and protein transcription. Then each left testis (T_L_) and each ovary (O) was snipped into pieces using clean dissection scissors. Hierarchical follicles, if present, were removed and not used for this experiment. The mass of each piece was recorded and then placed in to fresh culture medium containing: (1) 500 µl DMEM alone, (2) 0.25 µg/ml LH/FSH (44 LH: 1 FSH; National Hormone and Peptide Program, Torrance, CA) in DMEM, (3) 400 nM corticosterone (Sigma cat# C2505) and 0.25 µg/ml LH/FSH in DMEM, (4) 75 µg/ml 2-deoxy-D-glucose (2DG, Sigma cat# D8375) or 2.5 µg/ml ethyl 2-mercaptoacetate (MA, Spectrum Chemical Mfg. cat#E2833) and 0.25 µg/ml LH/FSH in DMEM. We hereafter refer to culture medium 1 as “basal” since it contains no treatment agents that would affect steroidogenesis. The ratio and concentration of LH/FSH we used has been previously defined as physiological and used successfully to stimulate sex steroid production (Kubokawa, Ishii & Wingfield, 1994; McGuire & Bentley, 2010a). Corticosterone is the native glucocorticoid in European starlings, and its plasma concentration is elevated during times of stress (Romero & Remage-Healey, 2000). 2DG is a pharmacological agent which potently inhibits glucose utilization (Wick et al., 1957). MA is a pharmacological agent which prevents tissues from oxidizing fatty acids (Bauche et al., 1983; Bernard, Mioskowski & Groscolas, 2002). 2DG/MA prevent the utilization of metabolic fuels, thus they were used here, in culture, to simulate the lack of available metabolic fuels experienced by European starlings under food-limited conditions. Concentration of total corticosterone used was within physiological range (Li et al., 2008; Romero & Remage-Healey, 2000). 2-DG and MA are pharmacological agents (Bauche et al., 1983; Wick et al., 1957).

All cultures were placed in a sealed incubator for a 4 h, high humidity incubation at 37°C immediately after placement of tissue. 100% oxygen was pumped into the chamber at 4L/min to allow for maximum respiration of cells. A time period of four hours was chosen to simulate a stress event of longer duration than typical capture restraint. The type of chronic stress that we are referring to would be experienced during an environmental perturbation (i.e., winter storm, habitat disturbance, microclimate change, human disturbance, temperature stress or food shortage). Corticosterone levels in birds are often chronically elevated during such events and these birds may also experience restricted access to food (Astheimer, Buttemer & Wingfield, 1992; Breuner & Hahn, 2003; Fernandez-Juricic, 2000; Jenni-Elermann et al., 2008; Ketterson & King, 1977; Lynn, Breuner & Wingfield, 2003; Morton, 2002; Wingfield & Ramenofsky, 1997). Although we aimed to mimic chronic stressors, the time period we chose may also be applicable to capture restraint stress paradigms as corticosterone can remain elevated for 30–180 min post-stimulus/CRS in a number of species (Wingfield, Vieck & Moore, 1992). Experimentally, the elevation of corticosterone in response to chronic stress is dampened only after days to weeks of exposure to stressors, thus a 4 h stress event would not elicit this phenomenon (Rich & Romero, 2005). A four hour culture is also applicable to metabolic stress, as birds have significantly reduced plasma glucose and triglycerides after a 2–12 h fast (Jenni-Elermann & Jenni, 1996). The *ex vivo* culture was not extended beyond 8 h so as to prevent artifacts associated with tissue death.

Following incubation, cultures were removed from the incubator, placed on ice and centrifuged at 1500 g 4°C. The supernatant was pipetted to a clean tube and stored at −20°C for sex steroid analysis using ELISA. The tissues were washed once each with DMEM and 0.01 M phosphate buffered saline, then stored at −20°C prior to analysis for GnIH and GnRH expression using semi-quantitative PCR with an endogenous control.

### Analysis of testosterone and estradiol secretion

The culture media supernatants from testes cultures were assayed for testosterone using ELISA (Cayman Chemical cat# 582701) and the culture media supernatants from ovary cultures were assayed for estradiol using ELISA (Cayman Chemical cat# 582251) according to the manufacturer’s instructions. These kits were developed specifically for use with tissue culture supernatants, and have specific instructions and general precautions for using this type of sample (see https://www.caymanchem.com/pdfs/582701.pdf and https://www.caymanchem.com/pdfs/582251.pdf for information on this and on specificity).

Briefly, standards and samples were incubated with testosterone or estradiol antiserum and testosterone or estradiol acetylcholinesterase tracer in mouse anti-rabbit IgG-coated microplate wells at room temperature for 2 h at 45 rpm. Blank and maximum binding (buffer only) wells were also prepared at this time. After washing, the wells were developed with Ellman’s reagent, 5, 5′-dithiobis-2-nitrobenzoic acid, in the dark for 60 min. Assays were performed in duplicate and were read at 415 nm on a microplate reader (BioRad, Model 680XR) at + 0, + 5, + 15 and + 30 min. Data were collected when maximum binding wells reached an absorbance within 0.3–1 A.U. All readings were corrected to the absorbance of the blank wells, then standards and samples were converted to percentages of maximum binding. A standard curve was drawn using log(agonist) v. response variable slope four parameter curve fit in GraphPad Prism 5.0 software. The concentrations of testosterone or estradiol in the culture media were interpolated from the appropriate standard curves.

Testosterone and estradiol concentrations from each culture were corrected to the amount of tissue in each culture. To control for differences in mass of gonad sections in each culture, testosterone and estradiol data are corrected to testes or ovary section mass measured just prior to placement in treatment cultures (pg testosterone or estradiol/mg tissue).

All samples were assayed in duplicate in a single assay. Assay characteristics were 20 pg/ml for estradiol and 6 pg/ml for testosterone. The intra-assay coefficient of variation for estradiol was 4.524 and for testosterone was 1.463.

### Analysis of GnIH and GnRH expression

Tissues from the February 5 cultures were analyzed for the effects of treatments on relative GnIH and GnRH mRNA expression using semi-quantitative PCR with an endogenous control. This method has been previously described (Foley, Leonard & Engel, 1993; McGuire, Kangas & Bentley, 2011; Spencer & Christensen, 1999). Briefly, RNA was isolated from tissue in each culture using TRIzol (Invitrogen cat# 15596018) according to the manufacturer’s protocol. RNA concentration, quality and contamination were assessed using NanoDrop and NanoDrop-1000 3.3.0 accessory software. RNA samples with 10 mm absorbance at wavelengths from 220–350 nm or 260/280 ratio of >1.6 were not used for further analysis. 1 ug total RNA from each tissue sample was then reverse transcribed using oligo(deoxythymidine)15 primer and M-MLV Reverse Transcriptase (Promega cat #s: C1101, M1701). Partial European starling GnIH and GnRH precursors were amplified from 500 ng cDNA by PCR using primers based on European starling GnIH and GnRH precursor cDNA sequences (respective GenBank accession #s EF486798, FJ514493): GnIH forward primer 5′-GGAAGAAAAGCAGAGGAGTCTC-3′, reverse primer 5′-TGGAGATCTCCCAAGCCTGT-3′; GnRH forward primer 5′-TCTCTCAGGCAGCAGGATGGA-3′, reverse primer 5′-5′-CTTTCTTCTGCCTTGTTCCTCC-3′. β-actin was also amplified from the cDNA and used as the endogenous control. All PCR amplifications were performed using a Taq polymerase kit (TaKaRa Ex Taq™; Takara Bio Inc., Shiga, Japan). Products were run on a 1.5% agarose gel and quantified by fluorescence of ethidium bromide under a UV Transilluminator (UVP Inc., Upland, CA) using a two-dimensional image analysis of the gel in Adobe Photoshop CS2. The intensity of each of the GnIH and GnRH signals was normalized to the B-actin internal control intensity of the same bird. The relative mRNA levels of GnIH and GnRH from the tissue in each culture (expressed as 0%–100% of B-actin) were then compared.

### Statistical analysis

Data were analyzed using one-way analysis of variance (ANOVA), followed by the post-hoc Tukey’s multiple comparison test if the ANOVA provided *P* < 0.05.

To determine if the treatments were any more or less effective in photostimulated birds versus photosensitive birds, 2-way ANOVA was performed with time and treatment as independent variables.

## Results

February 5, 2010 had a day length of 10 h 27 min. This daylength is less than the critical daylength of 11.5 h required for full photostimulation (and for the onset of ensuing photorefractoriness several weeks later) and was preceded by >6 weeks of short days. Birds were thus classified as photosensitive (pre-breeding) (Boulakoud & Goldsmith, 1995; Nicholls et al., 1987). Testes collected from European starling males had an average volume of 76.0 ± 22.5 mm^3^ (volume = 4/3π*a*^2^*b*, where *a* is half the width and *b* is half the length (long axis) of the testis). Ovaries collected from European starling females had an average ovarian mass of 6.2 ± 1.5 mg and all follicles were <1 mm in diameter.

April 7, 2010 had a day length of 12 h 52 min, which exceeds the critical daylength of 11.5 h required for photostimulation and for the resulting onset of photorefractoriness several weeks later. Birds were thus classified as photostimulated (breeding) (Nicholls et al., 1987). Testes collected from European starling males had an average testes volume of 502.0 ± 168.9 mm^3^ and ovaries collected from European starling females had an average ovarian mass of 30.2 ± 11.3 mg and had hierarchical follicles present.

**Figure 1:**
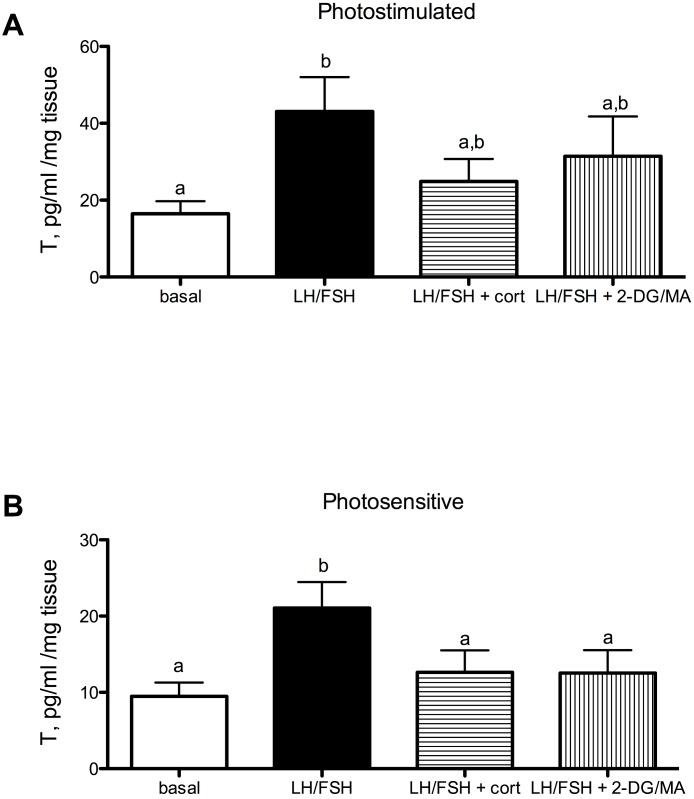
Seasonal differences in response to corticosterone and metabolic stress in European starling (*Sturnus vulgaris*) testes. (A) In testes collected from photostimulated male starlings, testosterone secretion is significantly increased in culture by incubation with LH/FSH compared to media alone. Corticosterone and the metabolic inhibitors 2DG/MA do not attenuate LH/FSH-stimulated testosterone levels in photostimulated starlings. (B) In testes collected from photosensitive male starlings, testosterone secretion is significantly increased in culture by incubation with LH/FSH compared to media alone. Corticosterone and the metabolic inhibitors 2DG/MA significantly attenuate LH/FSH-stimulated testosterone levels. Bars are mean testosterone secreted ± S.E.M. Different letters above columns indicate statistically significant differences.

Testosterone secretion in cultured European starling testes is significantly affected by culture treatments. However, the treatments are more effective prior to the breeding season compared to breeding birds (Fig. 1A: photostimulated, one way ANOVA *F*(5, 3) = 3.33, *p* < 0.048); Fig. 1B: photosensitive, one way ANOVA *F*(3, 5) = 10.67, *p* = 0.0005. When either photosensitive or photostimulated, testes are able to respond to culture with LH/FSH by increasing testosterone production significantly above basal levels (breeding: *p* < 0.05, prior to breeding: *p* < 0.001). Post-hoc comparison showed that when photosensitive, cultured testes exhibit significantly reduced testosterone secretion in the presence of corticosterone or metabolic inhibition (via 2DG/MA) compared to testes cultured in LH/FSH alone (corticosterone: *p* < 0.01; 2DG/MA: *p* < 0.01). Testes collected from photostimulated birds do not show reduced testosterone secretion in response to corticosterone or metabolic inhibition. Overall, there was an effect of season on the response to treatment (2-way ANOVA *F*(1, 3) = 12.66, *p* < 0.01).

**Figure 2:**
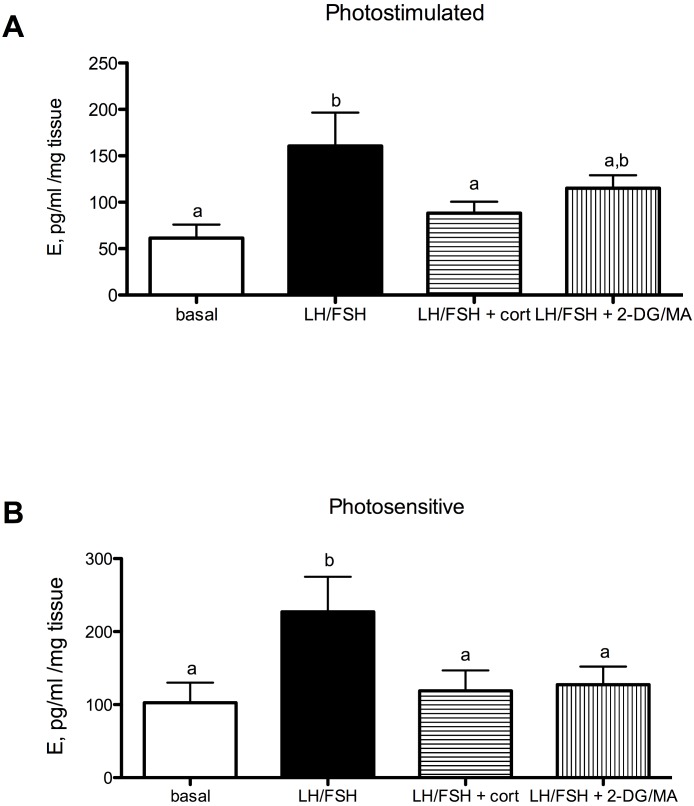
Seasonal differences in response to corticosterone and metabolic stress in European starling (*Sturnus vulgaris*) ovaries. (A) In ovaries collected from photostimulated female starlings, estradiol secretion is significantly increased in culture by incubation with LH/FSH compared to media alone. Corticosterone significantly attenuates LH/FSH-stimulated estradiol levels in photostimulated starlings. (B) In ovaries collected from photosensitive female starlings, estradiol secretion is significantly increased in culture by incubation with LH/FSH compared to media alone. Corticosterone and the metabolic inhibitors 2DG/MA significantly attenuate LH/FSH-stimulated testosterone concentrations. Bars are mean estradiol secreted ± S.E.M. Different letters above columns indicate statistically significant differences.

Estradiol secretion in cultured European starling ovaries is significantly affected by culture treatments in photosensitive (prior to breeding) and photostimulated (breeding) birds (Fig. 2A: photostimulated, one way ANOVA *F*(3, 5) = 6.6, *p* = 0.0046; Fig. 2B: photosensitive, one way ANOVA *F*(3, 5) = 7.31, *p* = 0.003. When photostimulated or photosensitive, ovaries are able to respond to culture with LH/FSH by increasing estradiol production significantly above basal levels (post-hoc comparison for photostimulated: *p* < 0.01; photosensitive: *p* < 0.01). Ovaries from photosensitive birds show significantly reduced estradiol secretion in the presence of corticosterone or metabolic inhibition (via 2DG/MA) compared to ovaries cultured in LH/FSH alone (corticosterone: *p* < 0.05; 2DG/MA: *p* < 0.05). Ovaries collected from photostimulated birds show reduced estradiol secretion in response to corticosterone only (corticosterone: *p* < 0.05). Overall, there was an effect of season on the response to treatment (2-way ANOVA *F*(1, 3) = 7.58, *p* < 0.02).

**Figure 3:**
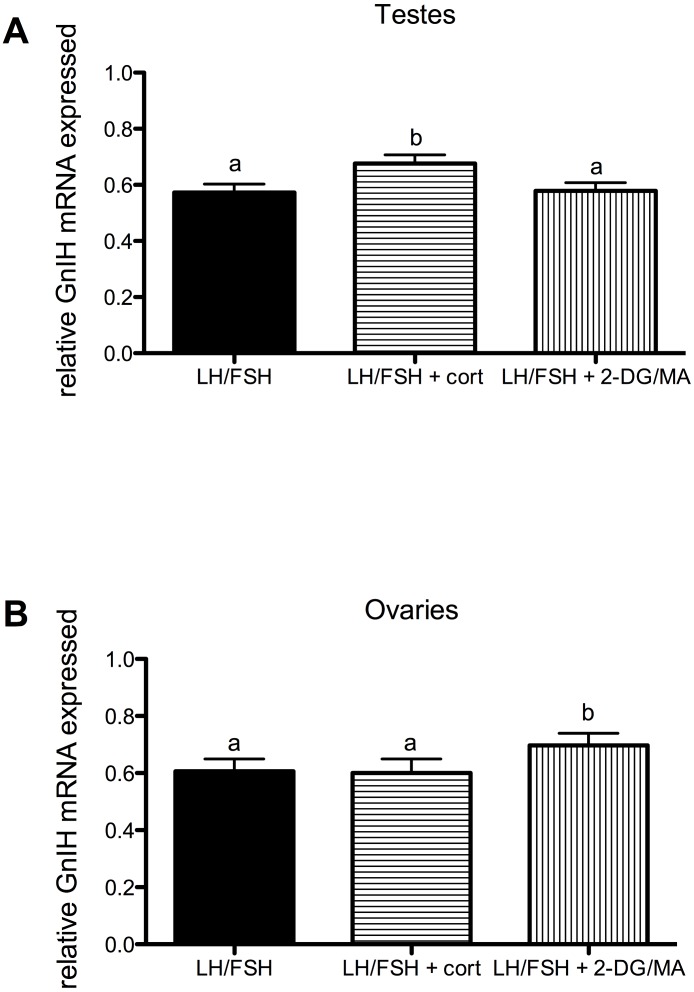
Stressors upregulate gonadal expression of GnIH mRNA in photosensitive birds. Expression of GnIH is significantly upregulated by corticosterone in the testes and by the metabolic inhibition via 2DG/MA in the ovary compared to LH/FSH stimulation alone. Bars are mean GnIH expressed ± S.E.M. Different letters above columns indicate statistically significant differences.

Corticosterone and the metabolic stressors 2DG/MA directly affect the expression of gonadal GnIH in gonadotropin-stimulated photosensitive European starling testes and ovaries (testes, Fig. 3A: one way ANOVA *F*(2, 5) = 12.70, *p* = 0.0018; ovaries, Fig. 3B: one way ANOVA *F*(2, 4) = 7.81, *p* = 0.013). In the testes, incubation with corticosterone significantly increases relative GnIH expression compared to LH/FSH-stimulated tissue (post-hoc comparison: *p* < 0.01). In the ovaries, incubation with the metabolic inhibitors 2-DG/MA significantly increases relative GnIH expression compared to LH/FSH-stimulated tissue (*p* < 0.05). GnIH expression did not differ between LH/FSH-treated ovarian tissue and LH/FSH+cort-treated ovarian tissue.

**Figure 4:**
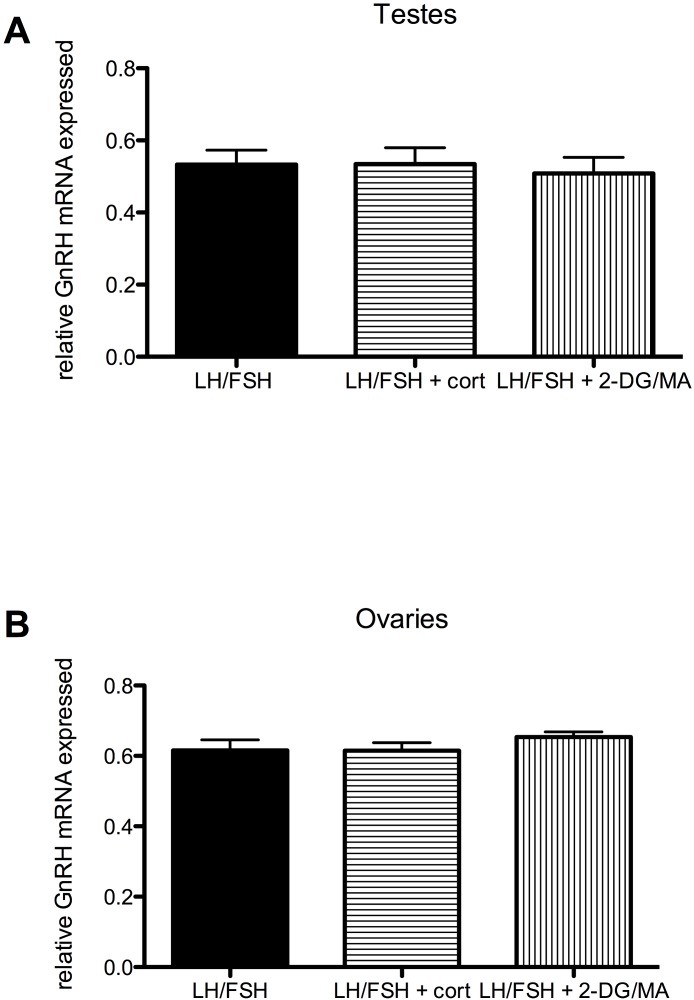
Stressors do not affect gonadal expression of GnRH mRNA in photosensitive birds. Expression of GnRH in the testes and ovaries is not significantly different in cultures treated with LH/FSH alone, LH/FSH +corticosterone or LH/FSH +2DG/MA. Bars are mean GnRH expressed ± S.E.M.

Stressors do not directly affect the expression of gonadal GnRH in gonadotropin-stimulated photosensitive European starling testes and ovaries (testes: one way ANOVA *F*(2, 5) = 1.00, *p* = 0.408; ovaries: one way ANOVA *F*(2, 5) = 2.794, *p* = 0.109). These data are shown in Fig. 4.

## Discussion

In addition to regulation from the brain, the gonads of European starlings (*Sturnus vulgaris*) are able to detect cues of stress directly and respond (in a season-specific manner) by modulating sex steroid secretion. The testes and ovaries of photosensitive (prior to breeding) European starlings show significantly reduced gonadotropin-stimulated testosterone and estradiol secretion when cultured with the glucocorticoid corticosterone, or with the metabolic inhibitors, 2DG/MA, compared to gonadotropin-stimulation alone. As birds become photostimulated and reach full reproductive condition, the testes no longer respond to corticosterone or 2DG/MA in culture with reduced testosterone secretion. The ovaries of photostimulated starlings do maintain their responsiveness to corticosterone at this time, but no longer respond to 2DG/MA in culture with reduced estradiol secretion.

What accounts for these seasonal and sex differences? Photosensitive birds are still making the ‘decision’ to breed, while photostimulated birds are already maximally stimulated by gonadotropins from the HPG axis. Supplementary cues (i.e., non-photic cues of the suitability of the future environment for breeding and rearing young, such as habitat quality, presence of mates, weather and stress) are much more important in the decision to delay or advance the onset of breeding, and appear to be less influential once birds have already committed to breeding (Ball, 1993; Dawson, 2008; Hinde & Steel, 1976; Wingfield & Sapolsky, 2003). This may be mediated physiologically in the gonads by a dampening of the responsiveness to stress hormones and metabolic stress during breeding. Additionally, recrudescence of the gonads represents a higher metabolic cost than simply maintaining large gonads once they have recrudesced (Vezina & Salvante, 2010), although in a study on wild birds housed indoors with *ad libitum* food, testicular growth was not thought to be energy-demanding (Caro & Visser, 2009). Thus when metabolic fuels are limited, the onset of gonadal growth (which would occur in photosensitive birds) is delayed, but maintenance of gonads (which would occur in photostimulated birds) is not. Interestingly, ovaries do continue to respond to corticosterone by locally reducing sex steroid secretion even while in breeding condition, while testes do not. This result might reflect the differing breeding strategies of males and females. Female songbirds do not initiate full follicle growth until conditions are favorable for laying (Morton, 2002). However, males will continue to support testosterone-stimulated behaviors/physiology even while stressed, so that fertilization opportunities are not lost (Ball & Ketterson, 2008; Beletsky, Orians & Wingfield, 1989).

Our data indicate that gonadal GnIH may be involved in mediating the season- and sex-specific responses to cues of stress. GnIH has been previously identified as a local inhibitor of testosterone production (McGuire & Bentley, 2010a) and GnIH is upregulated in the testes of photosensitive males in response to corticosterone and in the ovaries of photosensitive females in response to metabolic stress (via 2DG/MA). Thus the gonads are able to respond to cues of stress directly by up-regulating this hormone. In this way, the gonads of birds are able to fine-tune their steroid secretion and possibly optimize the onset and degree of gonadal recrudescence in response to their local environment. Alternatively, other hormones may be involved. An obvious difference between breeding and non-breeding birds is exposure level to circulating gonadotropins. LH and FSH may play a role in decreasing the responsiveness of the gonads to corticosterone and metabolic stress. Additionally, prior to the breeding season, photosensitive birds experience shorter days than photostimulated birds. Light cues are normally mediated through the brain and affect the gonads via hormones of the HPG axis (Dawson et al., 2001). However, it is possible the gonads can respond directly to cues of day length. Melatonin, a hormone secreted by the pineal gland at night (thus providing a proxy of day length), is detectable in the plasma in the same phase and duration as the brain (Maywood et al., 1993; Rollag, O’Callaghan & Niswender, 1978). Melatonin receptors are expressed in the gonads of birds and mammals, and melatonin appears to affect directly the physiological actions of the gonads (Ahmad & Haldar, 2010; Ayre & Pang, 1994; Frungieri et al., 2005; Murayama et al., 1997; Niedziela, Lerchl & Nieschlag, 1995). Melatonin may thus also play a role in the regulation of seasonal responsiveness to stress in the gonads. Additionally, glucocorticoid receptor may be seasonally regulated in the gonads of birds, much like amphibians (Denari & Ceballos, 2006). Alternatively, avian gonads may respond to stress through an additional pathway *in vivo* that circumvents the HPG axis: the neural brain-testicular pathway identified in rats (James, Rivier & Lee, 2008). The existence and seasonal regulation of this system is as yet unknown in birds and requires further investigation. The possible use of seasonal cycles of LH/FSH, melatonin or glucocorticoid receptors, however, does not exclude the use of seasonal cycles of gonadal GnIH. In fact, GnIH in the hypothalamus is upregulated independently by both melatonin and stress, and GnIH neurons express glucocorticoid receptors and melatonin receptors (Calisi, Perfito & Bentley, 2010; Ubuka et al., 2005). Thus there is a precedent for melatonin and corticosterone to influence GnIH directly.

The regulation and action of gonadal GnRH in songbirds requires further determination. However, we conclude here that GnRH mRNA is not upregulated in the gonads of photosensitive European starlings by the stress hormone, corticosterone, nor by the metabolic inhibitors, 2DG/MA.

In sum, the evidence provided by our model shows that the testes and ovaries of European starlings respond directly to chronically elevated corticosterone and metabolic stress independently of the brain by modulating testosterone and estradiol secretion. This modulation is season- and sex-specific: it occurs only while birds are deciding to breed, and it appears to involve the gonadal GnIH system. It is likely that this direct modulation of gonadal function in response to stress is most important prior to the onset of breeding because the gonads are not already maximally stimulated by components of the HPG axis and fully recrudesced. Taken together, our data indicate that photosensitive temperate songbird testes and ovaries are capable of responding directly to corticosterone and metabolic stress, in addition to GnRH-induced gonadotropin release, by increasing GnIH expression and decreasing testosterone and estradiol secretion. Thus, we suggest that the GnRH system is not the only pathway for integration of cues into a gonadal response.
